# Prognostic value of hyperlactatemia in infected patients admitted to intensive care units: a multicenter study

**DOI:** 10.5935/0103-507X.20220010-en

**Published:** 2022

**Authors:** Catarina Mendes Silva, João Pedro Baptista, Paulo Mergulhão, Filipe Froes, João Gonçalves-Pereira, José Manuel Pereira, Claudia Camila Dias, José Artur Paiva

**Affiliations:** 1 Centro Hospitalar e Universitário de Coimbra - Coimbra, Portugal.; 2 Grupo de Infeção e Sépsis - Lisboa, Portugal.; 3 Centro Hospitalar São João - Porto, Portugal.; 4 Hospital Pulido Valente, Centro Hospitalar Lisboa Norte - Lisboa, Portugal,.; 5 Hospital Vila Franca de Xira - Lisboa, Portugal.; 6 Department of Community Medical Information and Health Decision Sciences, Faculdade de Medicina, Universidade do Porto - Porto, Portugal.

**Keywords:** Hyperlactatemia, Infections, Lactate, Hospital mortality, Prognosis, Intensive care units

## Abstract

**Objective:**

To evaluate the influence of patient characteristics on hyperlactatemia in an infected population admitted to intensive care units and the influence of hyperlactatemia severity on hospital mortality.

**Methods:**

A *post hoc* analysis of hyperlactatemia in the INFAUCI study, a national prospective, observational, multicenter study, was conducted in 14 Portuguese intensive care units. Infected patients admitted to intensive care units with a lactate measurement in the first 12 hours of admission were selected. Sepsis was identified according to the Sepsis-2 definition accepted at the time of data collection. The severity of hyperlactatemia was classified as mild (2 - 3.9mmol/L), moderate (4.0 - 9.9mmol/L) or severe (> 10mmol/L).

**Results:**

In a total of 1,640 patients infected on admission, hyperlactatemia occurred in 934 patients (57%), classified as mild, moderate and severe in 57.0%, 34.4% and 8.7% of patients, respectively. The presence of hyperlactatemia and a higher degree of hyperlactatemia were both associated with a higher Simplified Acute Physiology Score II, a higher Charlson Comorbidity Index and the presence of septic shock. The lactate Receiver Operating Characteristic curve for hospital mortality had an area under the curve of 0.64 (95%CI 0.61 - 0.72), which increased to 0.71 (95%CI 0.68 - 0.74) when combined with Sequential Organ Failure Assessment score. In-hospital mortality with other covariates adjusted by Simplified Acute Physiology Score II was associated with moderate and severe hyperlactatemia, with odds ratio of 1.95 (95%CI 1.4 - 2.7; p < 0.001) and 4.54 (95%CI 2.4 - 8.5; p < 0.001), respectively.

**Conclusion:**

Blood lactate levels correlate independently with in-hospital mortality for moderate and severe degrees of hyperlactatemia.

## INTRODUCTION

Since its first description, hyperlactatemia has been related to morbidity and mortality.^(1)^ Increased lactate production in the early presentation of sepsis is frequently related to inadequate oxygen delivery and anaerobic glycolysis (type A hyperlactatemia).^(2)^ However, there are other causes not driven by hypoperfusion, such as drug effects, diabetic ketoacidosis, liver failure, malignancy and thiamine deficiency (type B hyperlactatemia).^(3)^ In septic shock, in addition to hypoperfusion, adrenergic-driven aerobic lactate production and impaired hepatic lactate clearance have also been suggested to contribute to hyperlactatemia.^(4)^

Previous studies investigated the impact of hyperlactatemia on admission mortality in patients admitted to the intensive care unit (ICU) and reported cumulative prevalence rates varying from 10 to 91%.^(5)^ On ICU admission, a higher lactate concentration within the normal reference range (relative hyperlactatemia) has been shown to be an independent predictor of hospital mortality in critically ill patients.^(6,7)^ Lactate levels between 2.0 - 3.9mmol/L in patients with suspected infection were associated with significant mortality, even in the absence of hypotension.^(8)^ Haas et al.^(9)^ found that the degree of hyperlactatemia was directly related to the severity of shock and to the mortality rate, which reached 80% in patients with lactate levels >10mmol/L.

Although hyperlactatemia on ICU admission has been shown to be a good prognostic marker, dynamic changes in lactate concentration have also proven to be independent predictive values.^(10)^ In fact, lactate level and lactate clearance were both useful targets in patients with suspected infection^(11)^ or septic shock defined by Sepsis-3^(12)^ in the emergency department, and in a recent study, 6-hour lactate level was more accurate than 6-hour lactate clearance in predicting 30-day mortality.^(13)^

Many factors confound the clinical use of lactate level. The most common in clinical practice are the use of catecholamines in septic shock patients, alkalosisinduced increases in glucose metabolism, lactate-buffered continuous hemofiltration, liver dysfunction, and lung lactate production.^(14)^ Medical literature is limited regarding how patient and disease characteristics may influence blood lactate values and whether those factors are determinants of outcomes.

The aims of this study were to evaluate the influence of patient characteristics on the presence of hyperlactatemia in an infected population on ICU admission and determine differences in outcomes and to investigate the association between the severity of hyperlactatemia and mortality.

## METHODS

### Study protocol

We carried out a *post hoc* analysis of the Infection on Admission to the ICU (INFAUCI) study, which was an observational, multicenter, prospective cohort study conducted in 14 Portuguese ICUs with data collected between 1st May 2009 and 31 December 2010.^(15)^ The study protocol was described elsewhere.^(15)^ Briefly, all adult patients (age ≥ 18 years) consecutively admitted during one followed until death or 6 months after ICU admission. The Hospital Research and Ethics Committee of *Centro Hospitalar São João* approved the study design. Informed consent was waived due to the observational nature of the study. For the purpose of this study, we analyzed arterial blood lactate levels on ICU admission in infected patients. The highest value of lactate within the first 12 hours of admission was recorded. Infection and sepsis criteria were identified at the time of admission to the ICU according to commonly used definitions accepted at the time of data collection, the Sepsis-2 definition^(16)^ According to this consensus, septic shock is defined as a state of acute circulatory failure characterized by persistent arterial hypotension unexplained by other causes. Data were collected on patient demographic and clinical characteristics, such as sex, age, Simplified Acute Physiology Score II (SAPS II), Sequential Organ Failure Assessment (SOFA) score, Charlson Comorbidity Index score,^(17)^ comorbidities, functional status, origin and diagnosis on admission, ICU length of stay (LOS) and hospital LOS. Primary outcome was in-hospital mortality. Arterial blood lactate was measured using a blood gas analyzer.

### Statistical analysis

Continuous variables SAPS II, SOFA score, Charlson Comorbidity Index score, ICU LOS and hospital LOS were dichotomized around the mean/median values found for all populations.^(15)^ Age was categorized into two groups: less than 65 years and 65 years and higher. A cutoff value of 2mmol/L was used to define hyperlactatemia. Hyperlactatemia was categorized into three groups according to severity: mild (2.0 - 3.9mmol/L), moderate (4.0 - 9.9mmol/L) and severe (>10.0mmol/L).

Categorical variables were described as absolute and relative frequencies, and continuous variables were expressed as median (percentile - P25 - P75) or mean ± standard deviation, according to data distribution. Comparisons between groups were performed with *t*-tests for independent samples, Mann-Whitney U tests or Kruskal-Wallis tests for continuous variables and Chisquared tests for categorical variables, as appropriate. Logistic regression was applied, and patient demographic and clinical characteristics were included in the univariate analysis considering the categories previously established: age (< 65, ≥ 65); SAPS II score (< 45, ≥ 45); SOFA score on admission (< 7, ≥ 7); Charlson Comorbidity Index score (< 4, ≥ 4); comorbidities (no/yes); infection source (pneumonia, tracheobronchitis, endovascular, intraemergency surgery, trauma); septic shock (no/yes); bacteremia (no, primary, secondary) and hyperlactatemia (< 2; 2 - 3.9; 4 - 9.9, ≥ 10). All variables with p < 0.05 in the univariate analysis were included in the final multivariate regression modeling (enter method). The associations between patient characteristics and primary outcome were assessed by the odds ratio (OR) with a 95% confidence interval (95%CI) estimated by the multivariate models developed, and goodness-of-fit was assessed by the Hosmer-Lemeshow statistic and test. Two models were fitted adjusting for other covariates by either the SAPS II or the SOFA score. Age, chronic liver disease, chronic respiratory disease and cancer were not included in the multivariate analysis concerning collinearity with the Charlson Comorbidity Index.

The Receiver Operating Characteristic (ROC) area under the curve (AUC) was used to evaluate the ability of blood lactate to predict in-hospital mortality, and ROC curves were compared using 95%CIs for the AUC. All reported p-values were two-sided, and the significance level was set at 5%. Data were statistically analyzed using IBM Statistical Package for the Social Science (SPSS),® version 24.0, software (IBM Corp., Armonk, NY, USA).

## RESULTS

### Population characteristics

**Figure 1:**
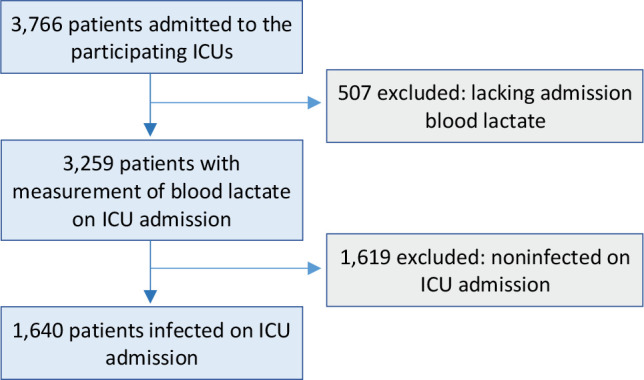
Selection of patients. ICU - intensive care unit.

A total of 3,766 patients admitted consecutively were included in the INFAUCI study. Blood lactate was measured on admission in 3,259 patients, with 1,640 patients being included in the infected group on ICU admission (Figure 1).

The median blood lactate level was 2.15mmol/L and significantly increased with age, SAPS II score, SOFA score on admission and Charlson Comorbidity Index score (Table 1S - Supplementary material). In regard to patients’ comorbidities, significantly higher blood lactate levels were observed in patients with chronic liver disease, immunosuppression and cancer. Septic shock and bacteremia were present in 49% and 20% of patients, respectively, and both were associated with higher blood lactate levels (p < 0.001). A longer ICU LOS was associated with significantly lower levels of lactate. Nonsurvivors had higher blood lactate levels than patients who survived. Intensive care unit and hospital nonsurvivors had median (P25 - P75) values of 3.4 (1.9 - 7.0) mmol/L and 3.0 (1.7 - 3.0) mmol/L, respectively, compared to 2.0 (1.2 - 3.1) and 1.9 (1.2 - 3.0) mmol/L in ICU and hospital survivors (p < 0.001).

In our cohort, 934 patients (57%) had hyperlactatemia on admission. Patient characteristics were evaluated in the univariate analysis for association with hyperlactatemia (Table 1), and significant variables were included in the multivariate logistic regression analysis adjusted by either SOFA score or SAPS II. A SAPS II ≥ 45, SOFA score ≥ 7, Charlson Comorbidity Index score ≥ 4, infections with intra-abdominal origin and presence of septic shock remained significantly associated with hyperlactatemia. Of these, the presence of septic shock was the factor that was most related to hyperlactatemia, with ORs (95%CIs) of 2.63 (1.99 - 3.45) and 2.72 (2.15 - 3.45), when adjusted for SOFA score and SAPS II, respectively.

**Table 1:** Univariate and multivariate logistic regression analysis of patient characteristics for hyperlactatemia (blood lactate level ≥ 2mmol/L; n = 934)

Variable	Univariate		Multivariate with SOFA		Multivariate with SAPS II	
	**OR (95%CI)**	**p value**	**OR (95%CI)**	**p value**	**OR (95%CI)**	**p value**
Immunosuppression§	1.28 (0.93 - 1.78)	0.132	-	-	-	-
Infection source						
Urological	1.00	-	1.00	-	1.00	-
Pneumonia	0.70 (0.45 - 1.09)	0.114	0.96 (0.57 - 1.64)	0.889	1.03 (0.60 - 1.66)	0.991
Tracheobronchitis	0.52 (0.29 - 0.92)	0.025	1.42 (0.67 - 3.01)	0.355	1.25 (0.61 - 2.54)	0.540
Endovascular	1.16 (0.65 - 2.08)	0.609	1.06 (0.49 - 2.27)	0.886	1.15 (0.54 - 2.44)	0.712
Intra-abdominal	1.63 (1.03 - 2.60)	0.039	2.09(1.14 - 3.82)	0.016	2.16 (1.22 - 3.84)	0.008
Skin and soft tissue	0.78 (0.44 - 1.40)	0.409	1.22 (0.59 - 2.51)	0.412	1.03 (0.53 - 2.12)	0.922
Neurological	0.48 (0.23 - 1.01)	0.053	0.98 (0.39 - 2.45)	0.980	0.83 (0.35 - 1.94)	0.664
Other	0.78 (0.33 - 1.82)	0.560	0.74 (0.28 - 1.96)	0.539	0.68 (0.25 - 1.79)	0.434
Diagnosis on admission						
Medical	1.00	-	1.00	-	1.00	-
Elective surgery	1.01 (0.54 - 1.87)	0.981	1.25 (0.53 - 2.99)	0.610	1.02 (0.44 - 2.38)	0.968
Emergency surgery	1.57 (1.25 - 1.98)	< 0.001	0.95 (0.64 - 1.41)	0.795	0.87 (0.59 - 1.26)	0.452
Trauma	0.48 (0.25 - 0.89)	0.020	0.49 (0.21 - 1.14)	0.096	0.57 (0.26 - 1.24)	0.153
Septic shock¶|	5.39 (3.67 - 7.92)	< 0.001	2.63 (1.99 - 3.45)	< 0.001	2.72 (2.15 - 3.45)	< 0.001
Bacteremia						
No	1.00	-	1.00	-	1.00	-
Primary	1.58 (0.97 - 2.55)	0.006	1.25 (0.62 - 2.51)	0.532	1.08 (0.54 - 2.14)	0.837
Secondary	1.91 (1.41 - 2.57)	< 0.001	1.42 (0.99 - 2.02)	0.051	1.32 (0.95 - 1.87)	0.103
Hosmer-Lemeshow p value	-		0.989		0.179	

SOFA - Sequential Organ Failure Assessment; SAPS II - Simplified Acute Physiology Score II; OR - odds ratio; 95%CI - 95% confidence interval.

* Reference group: Simplified Acute Physiology Score II < 45 points † reference group: Sequential Organ Failure Assessment score at admission < 7 points; ‡ reference group: Charlson Comorbidity Index < 4 points; § reference group: absence of disease; ¶ reference group: absence of septic shock (Sepsis-2 definition).^(16)^

### Hyperlactatemia severity

Patients presented with mild, moderate and severe hyperlactatemia in 57%, 34% and 9% of cases, respectively (Table 2). Higher degrees of severity of hyperlactatemia showed higher values of SAPS II (p < 0.001), SOFA score (p < 0.001) and Charlson Comorbidity Index score (p = 0.001), higher incidence of chronic liver disease as a comorbidity (p = 0.018), presence of septic shock (p < 0.001) and bacteremia (p < 0.001) (Table 2). On the other hand, the degree of hyperlactatemia was significantly inversely associated with chronic respiratory disease (p = 0.007). The hospital mortality rate showed a significant increase with the severity of hyperlactatemia, with values of 36%, 55% and 79% for mild, moderate and severe hyperlactatemia (p < 0.001), respectively. Intensive care unit and hospital LOS decreased with increased hyperlactatemia (p < 0.001). Figure 1S (Supplementary material) shows that this effect was more evident in the nonsurvivors. The difference in ICU and hospital LOS between survivors and nonsurvivors was statistically significant in all 3 categories of hyperlactatemia.

**Table 2:** Distribution of patient characteristics according to blood lactate category on admission

Variable	Blood lactate at admission (mmol/L) 2 - 3.9 4 - 9.9		≥ 10	p value*
	**(n = 532; 57%)**	**(n = 321; 34%)**	**(n = 81; 9%)**	
Female sex	210 (40)	124 (39)	30 (37)	0.905
Age				
< 65 years	266 (50)	141 (44)	35 (43)	0.168
≥ 65 years	266 (50)	180 (56)	46 (57)	
SAPS II	48 (16)	57 (18)	72 (19)	< 0.001†
SOFA score on admission	8 [3 - 16]	11 [4 - 18]	14 [9 - 20]	< 0.001‡
Charlson Comorbidity Index	5 (0 - 15)	5 (0 - 16)	7 (1 - 18)	0.001†
Comorbidities				
No comorbidities	54 (11)	27 (9)	6 (8)	0.526
Alcoholism	58 (11)	40 (13)	10 (12)	0.769
Chronic liver disease	32 (6)	23 (7)	12 (15)	0.018
Chronic kidney disease	47 (9)	32 (10)	8 (10)	0.846
Chronic respiratory disease	82 (16)	36 (11)	3 (4)	0.007
Chronic heart failure	54 (10)	40 (13)	10 (12)	0.547
Diabetes mellitus	121 (23)	70 (22)	23 (28)	0.464
Immunosuppression	58 (11)	39 (12)	11 (14)	0.727
Neurological disease	66 (13)	37 (12)	8 (10)	0.776
Cancer	217 (24)	81 (25)	28 (35)	0.127
Diagnosis on admission				
Medical	349 (66)	197 (61)	62 (77)	0.019
Elective surgery	19 (4)	3 (1)	1 (1)	
Emergency surgery	154 (29)	114 (36)	18 (22)	
Trauma	9 (2)	7 (2)	0 (0)	
Infection source				
Pneumonia	234 (44)	117 (37)	26 (32)	< 0.001
Tracheobronchitis	21 (6)	13 (4)	0 (0)	
Endovascular	30 (6)	19 (6)	15 (19)	
Intra-abdominal	160 (30)	121 (38)	25 (31)	
Skin and soft tissue	34 (6)	11 (3)	5 (6)	
Urological	19 (4)	30 (9)	6 (8)	
Neurological	11 (2)	5 (2)	2 (3)	
Other	10 (2)	4 (1)	1 (1)	
Septic shock	281 (54)	238 (75)	69 (86)	< 0.001
Secondary bacteremia	72 (14)	66 (22)	20 (27)	< 0.001
Appropriate initial antibiotics	205 (76)	136 (86)	28 (72)	0.020
Timing to antibiotic first dose				
≤ 1 hour	34 (26)	24 (32)	5 (29)	0.688
> 1 hour	95 (74)	51 (68)	12 (71)	
Length of stay				
ICU	9 (2 - 36)	8 (2 - 37)	3 (1 - 43)	< 0.001‡
Hospital	24 (6 - 94)	18 (2 - 81)	11 (2 - 89)	< 0.001‡
Hospital mortality	191 (36)	177 (55)	64 (79)	< 0.001

SAPS II - Simplified Acute Physiology Score II; SOFA - Sequential Organ Failure Assessment; ICU - intensive care unit.

* Chi-squared test; † ***t***-test for independent sample; ‡ Kruskal-Wallis test. Results expressed as n (%); mean (standard deviation) or median [P5 - P95].

### Effect of hyperlactatemia severity on mortality

The ROC curve for hospital mortality had an AUC of 0.64 (95%CI 0.61 - 0.67) for blood lactate values compared with 0.75 (95%CI 0.72 - 0.77) for SAPS II and 0.69 (95%CI 0.67 - 0.72) for SOFA score (Figure 2S - Supplementary material). In regard to hospital mortality, for a cutoff value of 2mmol/L for lactate levels, specificity was 51% and sensitivity was 69%, and for a cutoff value of 4mmol/L, specificity was 84% and sensitivity was 38%. Comparing the 95%CIs for the respective AUCs, we concluded that the combination of lactate with SOFA score did not significantly improve the performance of each variable alone, increasing the AUC to 0.71 (95%CI 0.68 - 0.74) for hospital mortality.

In a multivariate logistic regression, in-hospital mortality was correlated with moderate and severe hyperlactatemia but not with mild hyperlactatemia, with adjusted ORs of 2.07 (95%CI 1.47 - 2.92; p < 0.001) and 4.66 (95%CI 2.42 - 8.98; p < 0.001) for hospital mortality modeled with SOFA score in moderate and severe hyperlactatemia, respectively (Table 3). Similar values were obtained when the multivariate analysis was modeled with SAPS II, with ORs of 1.95 (95%CI 1.4 - 2.7; p < 0.001) and 4.54 (95%CI 2.4 - 8.5; p < 0.001) for moderate and severe hyperlactatemia, respectively.

**Table 3:** Univariate and multivariate logistic regression analysis of patient characteristics for hospital mortality

Variable	Univariate		Multivariate with SOFA		Multivariate with SAPS II	
	OR (95%CI)	p value	OR (95%CI)	p value	OR (95%CI)	p value
SAPS II ≥ 45 points*	5.21 (4.14 - 6.55)	< 0.001	-	-	3.77 (2.89 - 4.92)	< 0.001
SOFA score on admission ≥ 7 points†	3.31 (2.56 - 4.30)	< 0.001	2.12 (1.54 - 2.60)	< 0.001	-	-
Charlson Comorbidity Index ≥ 4 points‡	2.51 (2.01 - 3.13)	< 0.001	2.02 (1.54 - 2.63)	< 0.001	1.64 (1.27 - 2.14)	< 0.001
Septic shock§	2.44 (1.96 - 3.03)	< 0.001	1.27 (0.95 - 1.70)	0.099	1.35 (1.04 - 1.74)	0.024
Bacteremia						
No	1.00	-	1.00	-	1.00	-
Primary	1.93 (1.22 - 3.07)	0.005	1.71 (1.01 - 2.90)	0.044	1.27 (0.75 - 2.16)	0.374
Secondary	1.52 (1.15 - 2.01)	0.004	1.14 (0.83 - 1.58)	0.418	1.04 (0.75 - 1.43)	0.819
Hyperlactatemia (mmol/L)						
< 2	1.00	-	1.00	-	1.00	-
2 - 3.9	1.47 (1.15 - 1.87)	0.002	1.13 (0.85 - 1.51)	0.406	1.15 (0.86 - 1.55)	0.342
4 - 9.9	3.24 (2.46 - 4.27)	< 0.001	2.07 (1.47 - 2.92)	< 0.001	1.95 (1.40 - 2.70)	< 0.001
≥ 10	9.87 (6.64 - 17.27)	< 0.001	4.66 (2.42 - 8.98)	< 0.001	4.54 (2.42 - 8.50)	< 0.001
Hosmer-Lemeshow p value	-		0.536		0.999	

OR - odds ratio; 95%CI - 95% confidence interval; SAPS II - Simplified Acute Physiology Score II; SOFA - Sequential Organ Failure Assessment.

* Reference group: Simplified Acute Physiology Score II < 45 points † reference group: Sequential Organ Failure Assessment score at admission < 7 points; ‡ reference group: Charlson Comorbidity Index < 4 points; ¶ reference group: absence of disease; § reference group: absence of septic shock (Sepsis-2 definition.^(16)^

## DISCUSSION

In this large multicenter national study, clinical and epidemiological data from 14 Portuguese ICUs were collected, providing reliable and robust data that represent a picture of the country. We evaluated factors influencing hyperlactatemia on ICU admission of infected patients and its prognostic value. In some of the previous studies that used a single value of lactate on ICU admission, the first value measured on admission was selected;^(18-20)^ others used the highest value in the first 24 hours.^(21-23)^ In our investigation, the highest value in the first 12 hours of ICU admission was considered. We found that in a heterogeneous group of infected patients admitted to the ICU, hyperlactatemia was highly prevalent (57% of patients). In this group of critically ill patients, nearly half presented with septic shock. In the literature, the incidence of hyperlactatemia on admission in patients with severe infection has been shown to vary between 52% and 76%.^(18,24-26)^

Medical literature has demonstrated variable results related to the influence of hyperlactatemia on ICU or hospital LOS. Van den Nouland et al.^(27)^ found no differences in hospital LOS between patients admitted to the emergency department with lactate levels < 4mmol/L and ≥ 4mmol/L. In contrast, Chebl et al.^(28)^ observed that hospital LOS was longer for patients presenting to the emergency department with lactate levels > 4 mmol/L in comparison to 2 - 4mmol/L (10.4 ± 12.6 *versus* 8.1 ± 8.8 days), with a significantly higher mortality in the first group (40.7% *versus* 12%). In another study by Chebl et al.^(23)^ with a total of 16,447 patients admitted to the ICU, patients with lactate levels between 2 - 3.99mmol/L had a shorter hospital and ICU LOS than those with normal lactate levels; however, when restricted to survivors, differences in LOS were not statistically significant. Soliman et al.^(19)^ studied the relationship between blood lactate and LOS in a mixed ICU. They concluded that survivor patients with hyperlactatemia had a longer LOS than patients with normal lactate levels; on the other hand, hyperlactatemic nonsurvivors had a shorter LOS than nonsurvivors in the normal lactate level group. In our study, ICU and hospital LOS both decreased within classes of hyperlactatemia; however, after considering only survivors, an increase in ICU LOS was observed. The shorter LOS associated with higher lactate levels seems to be driven mainly by a higher mortality, as illustrated by the finding that the effect is limited to nonsurvivors. These findings highlight the importance of interpreting the effect of hyperlactatemia on LOS based on survival due to a competing risk bias.

The prognostic value of hyperlactatemia for mortality was first suggested by Broder et al. in 1964^(29)^ when they found that a lactate level > 4mmol/L in patients with shock from different causes was associated with death. Optimal cutoffs of single value lactate measurements in terms of prediction of outcome vary considerably in the literature, which in part may be justified by differences in the choice of lactate value for analysis, type of patients, severity on admission and outcome selection. Rivers et al.^(30)^ selected a serum lactate level of ≥ 4mmol/L to identify patients with severe sepsis or septic shock in the emergency department. In one study published in 2007, including patients infected on admission, an initial lactate level ≥ 4mmol/L was associated with a risk of in-hospital death three times higher than patients with a lactate level < 4mmol/L;^(31)^ in another study also published in 2007, including infected patients, a lactate level ≥ 4mmol/L showed an adjusted (for age and blood pressure) OR of 7.1 for 28-day inhospital death in comparison to patients with a lactate level < 2.5mmol/L.^(32)^ Of note, this cutoff of the lactate level (4mmol/L) was incorporated in the second edition of the Surviving Sepsis Campaign^(33)^ as an indicator of the need for fluid resuscitation in septic patients. In an analysis of the Surviving Sepsis Campaign database,^(25)^ serum lactate values greater than 4mmol/L were significantly associated with in-hospital mortality. In the group that had lactate measured within 6 hours, only patients with both a lactate value greater than 4mmol/L and hypotension maintained a statistically significant association after risk adjustment. However, in the Sepsis-3 consensus, the lactate cutoff for septic shock identification was changed from 4 to 2mmol/L, in order to improve sensitivity.^(34)^ In a recent retrospective study including 363 patients with sepsis and septic shock according to the Sepsis-3 definitions, a 6-hour lactate level of ≥ 3.5mmol/L was the optimal cutoff for 30day mortality.^(13)^ These results reinforce our findings that in-hospital mortality was correlated with hyperlactatemia above 4mmol/L but not with mild hyperlactatemia.

Mild hyperlactatemia and even values below the usual cutoff for hyperlactatemia of 2mmol/L have also been described as predictors of mortality. In a retrospective analysis using two septic shock cohorts,^(35)^ patients with initial lactate values between 1.4 and 2.3mmol/L had significantly greater 28-day mortality than patients who had baseline lactate values ≤ 1.4 mmol/L in both cohorts; however, the hazard ratio was not different from that obtained with blood lactate values between 2.3 and 4.4mmol/L (1.78 *versus* 1.65). Only for lactate values ≥ 4.4mmol/L was a significantly higher hazard ratio of 3.52 obtained.

Several studies have described an augmented risk of death related to an increase in blood lactate values.^(18,22,23,30)^ Ferreruela et al.^(36)^ observed an in-hospital mortality of 32.5% for critically ill patients in a mixed ICU for lactate concentrations during the ICU stay between 5 and 10mmol/L, which increased to 74.6% (p < 0.001) for hyperlactatemia > 10mmol/L. Haas et al.^(9)^ found an ICU mortality approaching 80% for patients with lactate levels > 10mmol/L, on at least one occasion in a retrospective study with 14,040 ICU patients. In our study, moderate (4 - 9.9mmol/L) hyperlactatemia was the lowest category predicting in-hospital mortality. Hospital mortality for lactate values > 10mmol/L reached 79%, which is in accordance with the previously mentioned studies.^(9,36)^

In the literature, values described for AUC of ROC curves of admission lactate and for in-hospital and 28day mortality have varied between 0.63 and 0.70 in patients with suspected infection,^(37)^ sepsis^(18,26,38)^ and septic shock.^(12,35)^ In our study, the AUC value for the ROC curve of lactate level for in-hospital mortality was in the range of previously described values (0.64). The AUC of the ROC curve of lactate was lower than that obtained with the scores for organ dysfunction, SAPS II and SOFA. The addition of lactate level to the SOFA score resulted in a marginally higher AUC of the ROC curve for hospital mortality (0.71).

Our study has several limitations. First, it is a post hoc analysis and for that reason not designed for this purpose. In the original protocol, the value of lactate collected was the highest in the first 12 hours of admission without any definition for the time of first lactate measurement or time to repeat measurements. However, our study was prospective, with a large sample size, and was conducted over a complete year. In the literature, most of the studies are retrospective, and some establish even higher intervals (24 hours) for defining lactate level at admission.^(7,22,23)^ Additionally, data on the possible pathophysiology or underlying mechanism of hyperlactatemia were not collected. There are multiple reasons for lactate elevation with different clinical relevance, and these confounders were not all categorized in this study; however, it was observed that in a general ICU population, hyperlactatemia is associated with mortality irrespective of underlying disease. Second, our study did not collect any data about lactate kinetics over time. However, lactate level and lactate clearance have both been shown to be useful targets in patients with suspected infection^(11)^ or septic shock.^(12)^ On the other hand, using a single value of lactate can be an easier and still valid method for predicting outcome. Third, septic shock patients were classified according to the Sepsis-2 definition^(15)^ accepted at the time of data collection, which has now been replaced by the Sepsis-3 definition.^(36)^ Although there was no direct correspondence between these two definitions, we maintained the septic shock subgroup in the analysis since it allowed us to identify the more seriously ill patients. Fourth, since this was a multicenter study, lactate measurements were not performed using similar equipment in the different institutions, which could induce some bias in the values obtained in different centers. However, this was a multicenter study with a large sample size allowing robust and statistically supported conclusions that can be generalized to other mixed ICUs.

## CONCLUSION

Hyperlactatemia on intensive care unit admission was present in more than half of a heterogeneous group of infected patients admitted to the intensive care unit and was a strong predictor of mortality. Blood lactate levels correlate independently with in-hospital mortality for moderate and severe degrees of hyperlactatemia.
